# The effect of angiotensin II on blood pressure in patients with circulatory shock: a structured review of the literature

**DOI:** 10.1186/s13054-017-1896-6

**Published:** 2017-12-28

**Authors:** Laurence W. Busse, Michael T. McCurdy, Osman Ali, Anna Hall, Huaizhen Chen, Marlies Ostermann

**Affiliations:** 1 Department of Medicine, Division of Pulmonary, Critical Care, Allergy, and Sleep Medicine, Emory University, Emory St. Joseph’s Hospital, 5665 Peachtree Dunwoody Road, Atlanta, GA 30342 USA; 2 Division of Pulmonary & Critical Care, Department of Medicine, University of Maryland School of Medicine, Baltimore, MD USA; 3 Department of Emergency Medicine, University of Maryland School of Medicine, Baltimore, MD USA; 4 Department of Critical Care, King’s College London, Guy’s & St Thomas’ NHS Foundation Hospital, London, SE1 7EH UK

**Keywords:** Angiotensin II, Distributive shock, Vasopressor, Vasoconstrictor, Cardiac arrest, Cardiogenic shock

## Abstract

**Background:**

Circulatory shock is a common syndrome with a high mortality and limited therapeutic options. Despite its discovery and use in clinical and experimental settings more than a half-century ago, angiotensin II (Ang II) has only been recently evaluated as a vasopressor in distributive shock. We examined existing literature for associations between Ang II and the resolution of circulatory shock.

**Methods:**

We searched PubMed, MEDLINE, Ovid, and Embase to identify all English literature accounts of intravenous Ang II in humans for the treatment of shock (systolic blood pressure [SBP] ≤ 90 mmHg or a mean arterial pressure [MAP] ≤ 65 mmHg), and hand-searched the references of extracted papers for further studies meeting inclusion criteria. Of 3743 articles identified, 24 studies including 353 patients met inclusion criteria. Complete data existed for 276 patients. Extracted data included study type, publication year, demographics, type of shock, dosing of Ang II or other vasoactive medications, and changes in BP, lactate, and urine output. BP effects were grouped according to type of shock, with additional analyses completed for patients with absent blood pressure. Shock was distributive (*n* = 225), cardiogenic (*n* = 38), or from other causes (*n* = 90). Blood pressure as absent in 18 patients.

**Results:**

For the 276 patients with complete data, MAP rose by 23.4% from 63.3 mmHg to 78.1 mmHg in response to Ang II (dose range: 15 ng/kg/min to 60 mcg/min). SBP rose by 125.2% from 56.9 mmHg to 128.2 mmHg (dose range: 0.2 mcg/min to a 1500 mcg bolus). A total of 271 patients with complete data were determined to exhibit a BP effect which was directly associated with Ang II. Subgroups (patients with cardiogenic, septic, and other types of shock) exhibited similar increases in BP. In patients with absent BP, deemed to be cardiac arrest, return of spontaneous circulation (ROSC) was achieved, and BP increased by an average of 107.3 mmHg in 11 of 18 patients. The remaining seven patients with cardiac arrest did not respond.

**Conclusions:**

Intravenous Ang II is associated with increased BP in patients with cardiogenic, distributive, and unclassified shock. A role may exist for Ang II in restoring circulation in cardiac arrest.

**Electronic supplementary material:**

The online version of this article (doi:10.1186/s13054-017-1896-6) contains supplementary material, which is available to authorized users.

## Background

Circulatory shock is a life-threatening condition with a high risk of multi-organ failure and death and a paucity of treatment options [1]. Catecholamines and vasopressin are often required to achieve an adequate blood pressure (BP), sometimes at the risk of adverse events, including peripheral and splanchnic ischemia, dysrhythmias, and organ dysfunction [2–5]. To date, no specific vasopressor has been shown to improve outcomes [6–8]. A 2015 meta-analysis by Zhou et al. concluded that other than the superiority of norepinephrine over dobutamine, no vasopressor outperformed any other with respect to mortality [9]. Two recent randomized controlled trials have renewed interest in angiotensin II (Ang II) for circulatory shock [10, 11]. In both of these studies, Ang II was hypothesized to increase mean arterial pressure (MAP) in patients with circulatory shock, as an adjunctive therapy to catecholamines and vasopressin standard-of-care therapy.

Ang II is a naturally occurring octapeptide that increases BP through various mechanisms, including vasoconstriction of peripheral vessels, potentiation of antidiuretic hormone (ADH) and adrenocorticotropin hormone (ACTH) release, and direct actions on postganglionic sympathetic fibers [12]. Following its discovery in the 1930s, Ang II has been administered to humans clinically and experimentally [13–18]. Considerable literature surrounds the clinical and experimental use of Ang II [19]. However, to our knowledge, no systematic evaluation has associated Ang II administration with improved BP in hypotensive states.

## Methods

### Study selection

We performed a review of all published reports of intravenous Ang II use in humans. We included full manuscripts published in English, excluding commentaries, conference presentations, duplicate reports, and animal studies.

The literature search was performed independently by two separate groups (MM and OA, MO and AH). Databases searched included PubMed, MEDLINE, Ovid, and Embase using the following search terms: “angiotensin” AND (“hypotension” or “shock”) AND “intravenous” AND “human,” and repeated using “hypertensin” or “angiotonin” as alternatives to “angiotensin.” Subsequently, we hand-searched the references of extracted papers for additional studies meeting inclusion criteria.

**Fig. 1:**
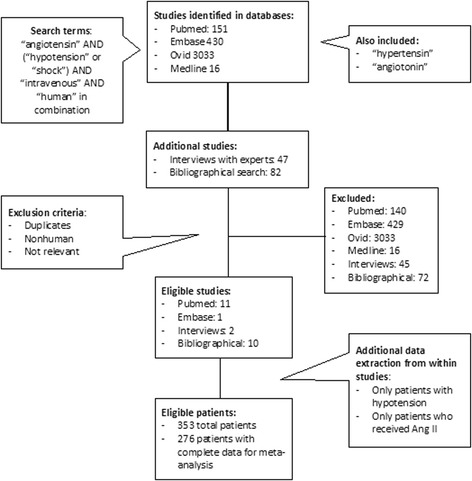
The search strategy utilized identified 3743 potential sources, from which 24 were selected for analysis. The search was executed by two independent groups of authors, with a fifth author as arbiter for study selection. Included studies described the blood pressure effects of human patients with hypotension who received Ang II as part of their therapy

Abstracts were reviewed by the same two groups of authors, who independently selected eligible studies. Circulatory shock was defined as MAP <65 mmHg or systolic blood pressure (SBP) <90 mmHg. For inclusion in the analysis, study subjects with circulatory shock were required to have received Ang II (+/- concomitant medications) and have post-Ang II BP documented. Disagreements on study selection were adjudicated by a fifth author (LB) not involved in the original literature search. The following data were extracted: study type, publication year, patient demographics, dose and duration of Ang II administration, dose and duration of other concomitant vasoactive medications, type of shock, and effects on BP, lactate and urine output (UOP). For studies reporting the use of Ang II in patients with and without hypotension, only patients with hypotension were analyzed. In studies reporting the effects of multiple vasoactive agents, only those patients receiving Ang II (+/- additional agents) were included. Figure 1 illustrates the literature selection process.

### Data analysis

Studies were analyzed for quantitative effects of Ang II on BP. The primary outcome was the change in MAP or SBP in association with Ang II. Individual patient data were extracted where available, allowing for standard error (SE) calculation. SE calculations did not include compiled data (i.e., 163 patients from the Angiotensin II for the Treatment of High Output Shock (ATHOS)-3 study for which no individual patient data were available). Results of each study were compiled and weighted according to the number of patients included in each study. Patients with missing or qualitative data were excluded from all calculations and described elsewhere. As a sub-analysis, we explored the association of Ang II in different types of shock, including cardiogenic, distributive, and other etiologies. Additionally, we performed an analysis of patients in whom BP was reported as zero or absent, which we defined as a condition of cardiac arrest. We performed sensitivity analyses by grading each study according to study type, and performed the original analysis for each category. Randomized, controlled trials (RCT) were graded as “A,” case controls as “B,” and case reports as “C.” Finally, we calculated changes in BP for the total population as well as for the septic shock cohort excluding ATHOS-3 patients, which represented nearly half of the total patient population.

## Results

The search in Ovid, MEDLINE, Embase, and PubMed retrieved 3743 potentially relevant abstracts, of which 95 articles were eligible for a more detailed evaluation. After full text evaluation, another 71 were excluded. Of the remaining 24 studies, two were RCTs, nine were case-control studies, and 13 were case reports. Details are shown in Fig. 1.

### Effects on blood pressure

In total, 353 patients with hypotension were administered Ang II, including 276 with complete data. Individual results of the selected studies, including dose ranges, median dose and interquartile dose ranges, are displayed in Table 1. Patients were hypotensive due to various etiologies, including cardiogenic (*n* = 38), distributive or septic (*n* = 225), or other causes (*n* = 90). For the purposes of this analysis, some forms of vasodilatory shock, such as neurogenic and medication-induced shock, were included as other causes of shock. In studies reporting changes in MAP (*n* = 218), BP rose by 23.4%, from an initial (i.e., prior to Ang II administration) weighted mean of 63.3 mmHg to 78.1 mmHg at an Ang II dose of 15 ng/kg/min to 60 mcg/min; SE of the mean (*n* = 45) was +/-14.0 mmHg. In studies reporting changes in SBP (*n* = 58), BP rose by 125.2% from 56.9 mmHg to 128.2 mmHg at a dose of 0.2 mcg/min to a 1500 mcg bolus; SE of the mean (*n* = 44) was +/-53.2 mmHg. One study (*n* = 8) reported an increase of diastolic blood pressure (DBP) by 20 mmHg from an initial average BP of 76/48 mmHg, at a dose range of 490 to 840 ng/min.

**Table 1:** Blood pressure effect of angiotensin II from entire cohort

Author (year)	Cases Total/+data	Type of shock			SBP ↑ (mmHg)	MAP ↑ (mmHg)	Dose range Median (IQR)
		Septic	Other	Cardiogenic			
Del Greco (1961) [25]	20/20	7	11	2	47.4		0.23–100 mcg/min 2.5 mcg/min (13.5)
Nassif (1963) [34]	14/13	6	6	2	106.9		7–1500 mcg bolus 14 mcg/min (32)
Wedeen (1963) [35]	15/7	1	3	11	81.1		1.5–36 mcg/min 10 mcg/min (6.5)
Beanlands (1964) [36]	17/0	0	0	17	^a^		1–36 mcg/min 2 mcg/min (2.25)
Udhoji (1964) [26]	12/6	4	5	3		34.3	^b^
Belle (1965) [37]	1/1	0	0	1	16.0		50–250 mg/day - -
Cohn (1965) [27]	6/6	6	0	0		29.7	^b^
Cohn (1965) [27]	22/22	0	22	0		22.1	0.3–60 mcg/min 14.1 mcg/min -
Singh (1966) [28]	25/0	25	0	0	^c^		4–12 mcg/min - -
Wallace (1967) [29]	7/7	0	7	0		22.9	0.75- mcg/min 1.5 mcg/min (0)
Sorensen (1986) [32]	8/0	0	8	0	^d^		^b^
Moore (1989) [33]	9/0	0	9	0	^e^		2.9 ng/kg/min - -
Geary (1990) [38]	1/1	0	0	1		30.0	6 mcg/min - -
Thacker (1990)	2/2	0	2	0		27.5	6–7 mcg/min 6.5 mcg/min -
Thomas (1991) [40]	1/0	1	0	0		^f^	5–20 mcg/min 10 mcg/min -
Jackson (1993) [20]	1/0	0	1	0	^g^		3–18 mcg/min 18 mcg/min -
Trilli (1994) [21]	1/1	0	0	1	24.0		8.5–9 mcg/min 8.5 mcg/min -
Ryding (1995) [41]	1/1	1	0	0		18.0	3.5–4.2 mcg/min - -
Newby (1995) [22]	1/1	0	1	0	30.0		0.8–2.2 mcg/min 22 ng/kg/min -
Wray (1995) [24]	1/0	1	0	0		^h^	8–22 mcg/min 8 mcg/min -
Tovar (1997) [23]	1/1	0	1	0	50.0		5–15 mcg/min 15 mcg/min -
Eyraud (1998) [30]	14/14	0	14	0	74.0		2.5 mcg bolus - -
Chawla (2014) [10]	10/10	10	0	0		6.0	15–20 ng/kg/min 20 ng/kg/min -
Khanna (2017) [11]	163/163	163	0	0		12.5	20–40 ng/kg/min 2.8 mg (5.8)
Total	353/276	225	90	38	71.3^i^	14.8^i^	

*SBP* systolic blood pressure, *MAP* mean arterial pressure, *IQR* interquartile range (in units similar to that study’s dose range)

^a^From 52.8 mmHg to > 100 mmHg in 13 of 18 patients

^b^Data unavailable

^c^From < 90 mmHg to > 90 mmHg

^d^From BP of 76/48 mmHg to DBP of > 68 mmHg

^e^20 mmHg increase from average SBP of 81.7 mmHg

^f^From 52 mmHg to > 100 mmHg

^g^From 50 mmHg to > 100 mmHg

^h^From < 80 mmHg to > 80 mmHg

^i^Weighted average

We performed a further analysis on all included patients to discern whether any of the observed BP effect could be associated with medications other than Ang II. As shown in Table 2, of all 353 patients described, 330 (93.4%) were thought to have realized a BP effect directly attributable to Ang II. Of these, 148 patients (41.9% of the total) received only Ang II. Of the 276 patients with complete data, 271 (98.1%) had BP effects attributed directly to Ang II. Nine patients were deemed to have realized a BP effect that could not be attributed to Ang II alone. These nine patients received various other pressors at varying doses (Table 2).

**Table 2:** Blood pressure effect of Ang II versus other vasoactive medications

Author (year)	Total number of cases	BP effects directly associated with Ang II	Cases with combined effect (Ang II + other)	Disclosed effects in cases with combined medication (Ang II + other)
Del Greco (1961) [25]	20	17	3	All three patients with initial BP 0/0 (cardiac arrest). 2/3 had no ROSC with addition of NE 54–200 mcg/min. 1/3 had ROSC with addition of NE at unknown dose, resulting in BP 100/0.
Nassif (1963) [34]	14	14	0	-
Wedeen^a^ (1963) [35]	15	7	0	Data available for only the 7 who responded. In these 7, NE was discontinued before Ang II started.
Beanlands (1964) [36]	17	13	4	All 4 patients received concomitant EPI 2–4 mcg/min.
Udhoji^a^ (1964) [26]	12	6	0	Data only available for 6 patients in whom Ang II was used alone.
Belle (1965) [37]	1	1	0	Co-administered with metaraminol (unknown dose) with hypotension on metaraminol alone. BP rose as direct result of Ang II
Cohn (1965) [27]	6	6	0	3 of 6 patients received NE before Ang II dose but NE was turned off prior to Ang II administration.
Cohn (1965) [27]	22	22	0	-
Singh (1966) [28]	25	25	0	-
Wallace (1967) [29]	7	7	0	-
Sorensen (1986) [32]	8	8	0	-
Moore (1989) [33]	9	9	0	-
Geary (1990) [38]	1	1	0	-
Thacker (1990)	2	0	2	1 patient received PHENYL (unknown dose), NE 3 mcg/kg/min, an EPI bolus (unknown dose) and DOPA 5 mcg/kg/min plus Ang II. 1 patient received DOPA 5 mcg/kg/min, EPI 4 mcg/min, PHENYL (unknown dose), and NE 3mcg/kg/min plus Ang II.
Thomas (1991) [40]	1	1	0	Co-administered with DOPA 6 mcg/kg/min, dobutamine (unknown dose), and NE 17 mcg/kg/min, but BP effect seen only after Ang II administration.
Jackson (1993) [20]	1	1	0	Co-administered with DOPA 3 mcg/kg/min and EPI 0.02 mcg/kg/min, but BP only responded after administration of Ang II.
Trilli (1994) [21]	1	1	0	Co-administered with DOPA 20mcg/kg/min, dobutamine (unknown dose), and NE 14.5mcg/min, but upon initiation of Ang II NE dose declined to 7mcg/min.
Ryding (1995) [41]	1	1	0	Co-administered with DOPA 4 mcg/kg/min, dobutamine (unknown dose), and NE at 28mcg/min, which were all titrated off after administration of Ang II and amrinone (unknown dose).
Newby (1995) [22]	1	1	0	Co-administered with DOPA 2.5 mcg/kg/min and NE 1 mg bolus, but BP only responded after Ang II administration.
Wray (1995) [24]	1	1	0	Co-administered with NE 8.3 mcg/kg/min and DOPA (low unknown dose), but BP only responded after administration of Ang II.
Tovar (1997) [23]	1	1	0	Co-administered with DOPA 4.9 mcg/kg/min and NE 60 mcg/min. BP only responded to addition of Ang II, which caused DOPA and NE to be turned off, both of which had to be restarted with Ang II cessation.
Eyraud (1998) [30]	14	14	0	-
Chawla (2014) [10]	10	10	0	Co-administered with VASO 0.02–0.08 u/min and NE 7.3–7.4 mcg/min. NE dose fell from a baseline 19.8 mcg/min upon initiation of Ang II.
Khanna^b^ (2017) [11]	163	163	0	Co-administered with a NE equivalent of 0.45 mcg/kg/min ranging down to 0.4 mcg/kg/min during 3 hours of Ang II administration, with positive BP effect seen at 3 hours, per study protocol.
Total	353	330	9	

*Ang II* angiotensin II, *BP* blood pressure, *ROSC* return of spontaneous circulation, *NE* norepinephrine, *EPI* epinephrine, *PHENYL* phenylephrine, *DOPA* dopamine, *VASO* vasopressin

^a^Patients from Wedeen et al. and 6 patents from Udhoji et al. were included in total number of patients who received Ang II for hypotension, but were not included in any quantitative analysis due to incomplete data. It cannot be determined whether these patients received Ang II alone

^b^NE equivalence established a priori as part of ATHOS-3 protocol

### Effects on BP by type of shock

Patients were further classified into subgroups based on the type of shock (Tables 3, 4, and 5). Of 38 patients with cardiogenic shock, 13 had complete data. SBP (*n* = 10) rose by 53.9 mmHg at an Ang II dose range of 1.2 mcg/min to a 350 mcg bolus, and MAP (*n* = 3) rose by 40.0 mmHg at a dose range of 6 mcg/min for one patient, with data unavailable for the other two. Of 225 patients with distributive or septic shock, 198 had complete data. SBP (*n* = 14) rose by 53.9 mmHg at a dose range of 0.2 mcg/min to a bolus of 120 mcg, and MAP (*n* = 184) rose by 13.3 mmHg at a dose range of 15 ng/kg/min to 4.2 mcg/min (with dose ranges available for only 174 patients). Of 90 patients with shock due to other etiologies (e.g., hemorrhagic shock, neurogenic shock, shock associated with chronic dialysis), 83 had complete data. SBP (*n* = 35) rose by 80.0 mmHg at a dose range of 0.8 mcg/min to a 1500 mcg bolus. MAP (*n* = 48) increased by 12.0 mmHg at a dose of 0.3 to 60 mcg/min.

**Table 3:** Cardiogenic shock

Author (year)	Cases	SBP ↑ (mmHg)	MAP ↑ (mmHg)	Dose range
Del Greco (1961) [25]	2	48.0		1.2–1.8 mcg/min
Nassif (1963) [34]	2	87.5		7–350 mcg bolus, 10.4–18.0 mcg/min
Wedeen (1963) [35]	11	57.0^a^		1.5–36.0 mcg/min
Beenlands (1964) [36]	17	^b^		1–36 mcg/min
Udhoji (1964) [26]	3		45.0^c^	N/A
Belle (1965) [37]	1	16.0		50–250 mg/day
Geary (1990) [38]	1		30.0	6 mcg/min
Trilli (1994) [21]	1	24.0		8.5–9.0 mcg/min
Total	38	53.9^d^	40.0^d^	

*SBP* systolic blood pressure, *MAP* mean arterial pressure

^a^Data from four responders. Seven nonresponders not included due to incomplete data

^b^From 52.8 mmHg to > 100 mmHg in 13 of 18 patients

^c^Data from two patients with complete data. A third patient had incomplete data

^d^Weighted average

**Table 4:** Septic shock

Author (year)	Number of cases	SBP ↑ (mmHg)	MAP ↑ (mmHg)	Dose range
Del Greco (1961) [25]	7	29.3		0.2–50.0 mcg/min
Nassif (1963) [34]	6	73.3		10–120 mcg bolus, 2–15 mcg/min
Wedeen (1963) [35]	1	110.0		11–21 mcg/min
Udhoji (1964) [26]	4		40.5	^a^
Cohn (1965) [27]	6		29.7	^a^
Singh (1966) [28]	25	^b^		4–12 mcg/min
Thomas (1991) [40]	1		^c^	5–20 mcg/min
Ryding (1995) [41]	1		18.0	3.5–4.2 mcg/min
Wray (1995) [24]	1		^d^	8–22 mcg/min
Chawla (2014) [10]	10		6.0	15–20 ng/kg/min
Khanna (2017) [11]	163		12.5	20–40 ng/kg/min
Total	225	53.9^e^	13.3^e^	

*SBP* systolic blood pressure, *MAP* mean arterial pressure

^a^Data unavailable

^b^From < 90 mmHg to > 90 mmHg

^c^From 52 mmHg to >100 mmHg

^d^From < 80 mmHg to > 80 mmHg

^e^Weighted averages

**Table 5:** Shock from other etiologies

Author (year)	Number of cases	SBP ↑ (mmHg)	MAP ↑ (mmHg)	Dose range
Del Greco (1961) [25]	11	61.8		0.8–130.0 mcg/min
Nassif (1963) [34]	6	129.2		25–1500 mcg bolus, 8.6–68.0 mcg/min
Wedeen (1963) [35]	^a^	115.0		2–18 mcg/min
Udhoji (1964) [26]	^b^		17.5	^c^
Cohn (1965) [27]	22		22.1	0.3–60.0 mcg/min
Wallace (1967) [29]	7		22.9	0.75–3 mcg/min
Sorensen (1986) [32]	8	^e^		^c^
Moore (1989) [33]	9	^f^		30 ng/kg/min
Thacker (1990)	2		27.5	6–7 mcg/min
Jackson (1993) [20]	1	^d^		3–18 mcg/min
Newby (1995) [22]	1	30.0		0.8–2.2 mcg/min
Tovar (1997) [23]	1	50.0		5–15 mcg/min
Eyraud (1998) [30]	14	74.0		2.5 mcg bolus
Total	90	80.0^g^	12.0^g^	

*SBP* systolic blood pressure, *MAP* mean arterial pressure

^a^Two patients with complete data. One with no data was a nonresponder

^b^Includes only two patients with data

^c^Data unavailable

^d^From 50 mmHg to >100 mmHg

^e^From BP of 76/48 to DBP of >68

^f^20 mmHg increase from average SBP of 81.7 mmHg

^g^Weighted averages

### Effects on organ perfusion

Only seven of the 24 studies commented on lactate or UOP. Chawla et al. noted no change in lactate or UOP in the Ang II group [10], while Khanna et al. made no reference to either serum lactate or UOP [11]. None of the case controls or case studies reported serum lactate values, but five studies did comment on UOP [20–24]. Both Thomas et al. and Wray et al. describe anuria which persisted after the initiation of Ang II, while Jackson et al., Newby et al. and Tovar et al. report an improvement in UOP. Scarcity of data prevented any meaningful conclusion of the effect of Ang II on organ perfusion.

### Special considerations and sensitivity analysis

Significant heterogeneity in study design and lack of a comparator in most studies prevented a thorough meta-analysis, though in 21 of 24 studies, we were able to extract patient-specific data. Additionally, the quality of the data presented within the various studies varied widely. (Additional file 1: Table S1) Two studies [10, 11] were graded as “A,” nine studies [25–33] were graded as “B,” and 13 studies [20–24, 34–41] were graded as “C.” In the two RCTs (*n* = 173), an increase in MAP of 12.1 mmHg was observed, with a concomitant decrease in norepinephrine doses [10, 11]. In nine studies graded “B” (*n* = 75), SBP and MAP increased by 58.7 mmHg and 25.1 mmHg, respectively. In 13 studies graded as “C” (*n* = 28), SBP and MAP rose by 86.6 mmHg and 25.8 mmHg.

Four studies included 18 patients described as having a SBP or MAP of 0 mmHg. While the studies did not overtly label these patients as having a cardiac arrest, we classified them as such. Among this cohort, complete data were available for 13 patients, and Ang II administration was associated with an increase in SBP of 107.3 mmHg, with a range of 0 to 250 mmHg (Additional file 2: Table S2). Two of these 13 patients did not have return of spontaneous circulation (ROSC); and among the other five patients with incomplete data, two were nonresponders. When excluding all 18 cardiac arrest patients from the primary analysis, Ang II was found to increase SBP and MAP in all patients with circulatory shock by 58.0 mmHg and 14.8 mmHg, respectively (Additional file 3: Table S3).

Of 276 patients for whom complete data existed, the recently published ATHOS-3 RCT accounted for 163 patients (59.3% of the total cohort), and comprised 82.3% of the 198 patients with septic or distributive shock and complete data. Following exclusion of the ATHOS-3 cohort, we calculated BP change for the remaining cohort with complete data (*n* = 113), as well for the septic patients (*n* = 35), (Additional file 4: Table S4 and Additional file 5: Table S5). SBP and MAP rose by 70.0 mmHg (*n* = 58) and 21.7 mmHg (*n* = 55), respectively, in the total cohort. In septic patients, SBP and MAP rose by 53.9 mmHg (*n* = 14) and 19.9 mmHg (*n* = 21).

Seventy-one of the total of 353 (20.1%) patients failed to respond adequately to Ang II, defined as either no response in BP or an increase in BP to a level below a MAP of 65 mmHg or SBP 90 mmHg. Of 71 nonresponders, four had cardiac arrest. Fifty-two nonresponders had septic shock, including 48 identified in the ATHOS-3 study. Thirteen patients with cardiogenic shock failed to respond to Ang II, as did six with shock of other etiologies.

## Discussion

Administration of Ang II in patients with hypotension appears to be associated with an increase in BP. The effect was seen in patients with shock of different etiologies, including distributive, cardiogenic, and other subtypes. Doses ranged from 15 ng/kg/min to bolus therapy of 1500 mcg. Ang II also appears to be associated with an increase in BP in patients with angiotensin-converting enzyme (ACE) inhibitor overdose and in patients on chronic dialysis. The pressor effect of Ang II was variable, ranging from a decrease in MAP of 8 mmHg to an increase in SBP of 250 mmHg in all patients. Nonetheless, the weighted means remained fairly consistent among the total cohort and subgroups (Table 6). Sensitivity analyses following exclusion of ATHOS-3 and cardiac arrest patients yielded results similar to the primary analyses. Sensitivity analyses according to the quality of studies were not substantially different, but did reflect a trend toward more robust results in poorer quality studies.

**Table 6:** Summary of results

Cohort	From	Cases with complete data	Increase in SBP (mmHg)	Increase in MAP (mmHg)
All patients	Table 1	276	71.3	14.8
Cardiogenic	Table 2	38	53.9	40.0
Septic	Table 3	225	52.3	13.3
Other	Table 4	90	80.0	12.0
Cardiac arrest patients	Table 5	18	107.3	
"A" studies	Additional file 1: Table S1	173		12.1
"B" studies	Additional file 1: Table S1	75	58.4	25.1
"C" studies	Additional file 1: Table S1	28	86.6	25.8
All patients except cardiac arrest	Additional file 2: Table S2	264	58.0	14.8
All patients except ATHOS-3	Additional file 3: Table S3	113	70.0	21.7
All septic patients except ATHOS-3	Additional file 4: S4	63	52.3	19.9
Standard error			17.7	8.7

*SBP* systolic blood pressure, *MAP* mean arterial pressure

The two included RCTs demonstrated an improvement of BP by only 6.0 mmHg (8.7%) and 12.5 mmHg (18.9%) [10, 11]. However, this was achieved in the context of a concomitant decrease in catecholamine doses [10] or as part of a protocolized BP endpoint which included titration of Ang II to a target MAP increase of 10 mmHg [11]. As such, comparisons to other studies is challenging. Nonetheless, the catecholamine-sparing effect and BP effect in these studies are consistent with the conclusion contained herein.

The consistent BP increase from Ang II may be explained in teleological terms. Ang II is a molecule innate to human physiology and, along with catecholamines and vasopressin, helps maintain BP throughout a variety of conditions. Its widely described effects include direct vasoconstriction of peripheral vessels, potentiation of water reabsorption as part of the renin-angiotensin-aldosterone system (RAAS), and interaction with other endogenous pressors (catecholamines and vasopressin). It is produced via multiple pathways throughout various tissue types and has important autocrine and paracrine functions, including participation in the cellular lifecycle and immune regulation [42]. The ubiquity of Ang II, its precursors and derivatives, and its receptors underlie a complex, highly evolved, homeostatic system of BP regulation. For example, RAAS activation during longstanding cardiovascular disease increases BP to maintain perfusion, but it also potentiates cardiac remodeling to withstand this increased BP [43]. Ang II also helps regulate and maintain glomerular filtration, especially during periods of reduced renal perfusion [44, 45].

Since its discovery in the 1930s, Ang II has been reported in over 31,000 humans in 1124 studies [19]. In the mid-1960s, intravenous Ang II was administered to pregnant women for preeclampsia evaluation [15, 16]. It has also been used in patients with a multitude of diseases, including circulatory shock [17, 24, 40, 41, 46] and ACE inhibitor overdose [20–22]. The 2014 ATHOS RCT highlighted the catecholamine-sparing effect of Ang II in patients with distributive shock [10]. Results of the recently completed ATHOS-3 study have confirmed its beneficial effect and safety in patients with distributive shock refractory to conventional vasopressor therapy [11].

Importantly, Ang II has been implicated as contributing to cardiac pathophysiology. Activation of the RAAS is associated with ventricular remodeling both at the site of infarct and remotely after myocardial ischemia [47]. Ang II also increases sympathoexcitation, which leads to hypertension and other cardiovascular diseases [48], and it upregulates peptides causing fibrogenesis, chemotaxis of fibroblasts, and scar formation in the healing heart [49]. ACE concentrations within myocardial tissue correlate with tissue collagen levels, and processes like fibrogenesis may cause further ACE accumulation in areas of cardiac remodeling [47]. Due to its ability to mitigate adverse myocardial remodeling, ACE inhibition has become the standard of care for patients with congestive heart failure [50]. Despite inhibition, however, patients with congestive heart failure can have elevated Ang II levels, which are associated with worse morbidity and mortality [51, 52]. Long-term effects of intravenous Ang II on cardiovascular health are unknown.

The cardiogenic shock population deserves special mention because of its exclusion from the two aforementioned RCTs [10, 11]. Scant data describe ACE or Ang II levels in patients with cardiogenic shock, though increased levels of Ang II and downregulation of Ang II receptors have been reported in other forms of shock [42, 53, 54]. Patients with cardiogenic shock may have a relative deficiency of Ang II, based on similar pathogenic mechanisms. While many studies in this review predate the widespread use of ACE inhibitors, the effect of Ang II in the five patients evaluated in the ACE-inhibition era [i.e., occurring after practice changes as a result of Cooperative North Scandinavian Enalapril Survival Study (CONSENSUS) and Studies of Left Ventricular Dysfunction (SOLVD) trials] suggest a potential role in patients on ACE inhibition who present with cardiogenic shock [55, 56]. Notably, four of the five patients had premorbid ACE inhibitor use, while information was unavailable for the remaining patient. The profound effect seen in these five patients (83.9% increase from baseline BP) suggests that administration of exogenous Ang II may restore the RAAS effect on BP. While exposure to angiotensin receptor blocker (ARB) therapy was noted in in the study by Khanna et al., the impact of ARB therapy was not specifically investigated [11]. In fact, the effect of Ang II on patients pre-treated with ARB therapy may be difficult to predict owing to heterogeneity in pharmacodynamics and pharmacokinetics of ARB therapy [56–58]

Eighteen patients included in this analysis had an initial BP of 0 mmHg, suggesting a condition of cardiac arrest. The pressor effect seen in these patients ranged from no change in BP (no ROSC) to an increase of 250 mmHg. The mean effect was 107.3 mmHg in patients with ROSC. Although cardiac arrest in these patients cannot be verified, their successful resuscitation suggests a potential role of Ang II to achieve ROSC. Ang II has not been described specifically for cardiac arrest, but a 2016 porcine cardiac arrest study examined Ang II levels with and without ACE inhibitor pretreatment [59]. Angiotensin (1–7) and Ang II levels increased in all animals after ROSC, though the effect on Ang II was muted in the ACE inhibitor-treated group and survival did not differ between groups. It should be noted that Wang et al. hypothesize that ACE inhibition, rather than an increase in Ang II, is beneficial in a cardiac arrest situation due to amelioration of ischemia-reperfusion injury in the myocardium as shown in a pig model. Nonetheless, the manipulation of the RAAS system in cardiac arrest may be a potential target for future investigation, as ischemia-reperfusion injury would be predicted to happen in all therapies for resuscitation, including catecholamines (which are the current standard of care). The mechanisms surrounding a potential benefit of Ang II in cardiac arrest are only speculative but may include: (a) maintenance of adequate systemic BP to vital organs [60], (b) manipulation of intracellular calcium levels leading to increased inotropy [61, 62], (c) potentiation of catecholamines [63, 64], and (d) increased afterload for enhancement of coronary perfusion [65]. Our observations are hypothesis-generating and require further properly constructed RCTs to elucidate the role of Ang II in cardiac arrest.

To our knowledge, this is the only analysis to thoroughly review the association of Ang II with increases in BP in patients with circulatory shock. The systematic selection of studies, the reproducibility of search results, and careful extraction of data support the comprehensiveness of the analysis. The consistent observed pressor effect over a wide variety of patients with different clinical conditions over a 66-year period (1961–2017) adds credence to the generalizability of the results.

This analysis has a number of weaknesses, including the lack of quality data. Only two of 24 studies analyzed were RCTs, with other studies being case-control and case-study formats. As such, the lack of a comparator to Ang II limits our ability to conclude causality. Of the 353 patients included in the analysis, 163 were from one source, potentially leading to a skewed effect [11]. However, this one source was a well-designed RCT, lending credibility to the results. Additionally, the risk of publication bias exists, as we were unable to identify any papers reporting lack of effect in patients treated with Ang II. Most studies were designed to address research questions either unrelated or tangentially related to the pressor effect of Ang II. Nonetheless, included in this analysis are 51 patients in the two RCTs who received Ang II but did not respond, as well as 30 nonresponders in the case-control analyses and case studies. The inclusion of nonresponders, in our opinion, mitigates publication bias. The quality and heterogeneity of data (e.g., BP variably reported as MAP, SBP, or DBP, and some BP reported graphically or qualitatively as “normal”) limit robust quantitative analysis. However, this heterogeneity was expected, and the a priori primary endpoint was designed specifically to account for this. We chose a simple, easy-to-identify, frequently reported endpoint (BP) so as to be able to include as many data points as possible. The specific effect of Ang II may also be questioned, as many studies described its BP effect in association with the co-administration of other vasoactive medications (e.g., norepinephrine, epinephrine, and amrinone). However, this approach mirrors clinical management of shock, in which multiple agents are often used. Moreover, Ang II was evaluated in the ATHOS-3 study as a therapeutic adjunct to other drugs, including catecholamines and vasopressin, which may add legitimacy to our results [11]. In both RCTs included herein, doses of catecholamines and vasopressin were controlled, allowing for clear elucidation of the isolated effect of Ang II. In further analysis, the observed BP effects in a vast majority of the patients analyzed herein can be attributed to or associated with Ang II alone, with only a handful of patients (*n* = 9) responding to a combination of medications including Ang II. Much of the analysis contained herein is derived from fairly old data. Ten of the 24 studies were published in the 1960s, while two were published in the 1980s, and ten in the 1990s. Only two studies (the two RCTs) have been published since the year 2000. As such, the standard of care for many disease states may be substantially different today. For example, ARB therapy is only described in the study by Khanna et al., despite the common use of this class of medication currently. ARB therapy may significantly affect the clinical response to Ang II, the effects of which remain unknown. Finally, as part of our comprehensive search, we deliberately omitted non-English language sources, and may have inadvertently omitted relevant English-language sources.

## Conclusions

In conclusion, Ang II appears to be associated with an increase in BP in patients with circulatory shock. The effect was robust, with increases in excess of 70 mmHg in SBP and 14 mmHg in MAP from the entire patient cohort, and remained observable in shock of various etiologies, including distributive, cardiogenic, and other circulatory shock. Observations in patients with cardiogenic shock and with ACE inhibitor use suggests a potential role for Ang II in patients with ACE depletion, either from acute ACE inhibitor overdose or as part of chronic heart disease management. Moreover, a role for Ang II in patients with cardiac arrest may warrant further exploration. More research, including well-designed randomized, controlled analyses are required to answer these questions.

## Additional files

Additional file 1: Table S1. Data analysis categorized by quality of study, describes primary analysis broken into subgroups based on a priori categorized study quality, which rates RCTs as “A,” case controls as “B,” and case reports as “C”. (DOCX 17 kb)

Additional file 2: Table S2. Cardiac arrest, describes results of the Ang II effect associated with all patients identified by the authors as being in a state of cardiac arrest (i.e., BP of 0/0). (DOCX 13 kb)

Additional file 3: Table S3. Results after removal of all cardiac arrest patients, describes primary analysis after removal of all patients identified by the authors as being in a state of cardiac arrest (i.e., BP of 0/0). (DOCX 15 kb)

Additional file 4: Table S4. Results with patients from Khanna et al. Rrmoved, describes primary analysis after exclusion of the patients from Khanna et al., which represent a proportionally large amount of the total patients included in the analysis. (DOCX 17 kb)

Additional file 5: Table S5. Septic shock results removing patients from Khanna et al., describes the analysis of Ang II n the septic shock sub-group after exclusion of the patients from Khanna et al., which represent a proportionally large amount of the total patients included in the analysis. (DOCX 13 kb)

## Abbreviations

ACE: Angiotensin-converting enzyme
ACTH: Adrenocorticotropin hormone
ADH: Antidiuretic hormone
Ang II: Angiotensin II
ARB: Angiotensin receptor blocker
ATHOS: Angiotensin II for the Treatment of High Output Shock
BP: Blood pressure
CONSENSUS: Cooperative North Scandinavian Enalapril Survival Study
DBP: Diastolic blood pressure
MAP: Mean arterial pressure
RAAS: Renin-angiotensin-aldosterone system
RCT: Randomized, controlled trial
ROSC: Return of spontaneous circulation
SBP: Systolic blood pressure
SE: Standard error
SOLVD: Studies of Left Ventricular Dysfunction
UOP: Urine output
