# Supplementary material for: The effect of angiotensin II on blood pressure in patients with circulatory shock: a structured review of the literature
Source: Crit Care. 2017 Dec 28;21:324. doi: 10.1186/s13054-017-1896-6 (PMC5745607; doi:10.1186/s13054-017-1896-6)
Supplement: Supplementary file 1 — Data analysis categorized by quality of study, describes primary analysis broken into subgroups based on a priori categorized study quality, which rates RCTs as “A,” case controls as “B,” and case reports as “C”. (DOCX 17 kb) [file 13054_2017_1896_MOESM1_ESM.docx]

| **Table S1. Data Analysis Categorized by Quality of Study** | | | | | |
| --- | --- | --- | --- | --- | --- |
| **Author** | **Cases** | **With complete data** | **Increase in SBP** | **Increase in MAP** | **Dose Range** |
| *A Rated* |  |  |  |  |  |
| Chawla | 10 | 10 |  | 6.0 | 15-20 ng/kg/min |
| Khanna | 163 | 163 |  | 12.5 | 20-40 ng/kg/min |
| **Total** | **173** | **173** |  | **12.1***^a^* |  |
| *B Rated* |  |  |  |  |  |
| Del Greco | 20 | 20 | 47.4 |  | 0.23-100 mcg/min |
| Udhoji | 12 | 6 |  | 34.3 | *^b^* |
| Cohn (AIM) | 6 | 6 |  | 29.7 | *^b^* |
| Singh | 25 | 0 | *^c^* |  | 4-12 mcg/min |
| Wallace | 7 | 7 |  | 22.9 | 0.75=3 mcg/min |
| Eyraud | 14 | 14 | 74.0 |  | 2.5 mcg bolus |
| Cohn (JCI) | 22 | 22 |  | 22.1 | 0.3-60 mcg/min |
| Sorensen | 8 | 0 | *^d^* |  | *^b^* |
| Moore | 9 | 0 | *^e^* |  | 30ng/kg/min |
| **Total** | **123** | **75** | **58.4***^a^* | **25.1***^a^* |  |
| *C Rated* |  |  |  |  |  |
| Nassif | 14 | 13 | 106.9 |  | 7-1,500 mcg bolus, 5.3-68 mcg/min |
| Wedeen | 15 | 7 | 81.1 |  | 1.5-36 mcg/min |
| Beenlands | 17 | 0 | *^f^* |  | 1-36 mcg/min |
| Belle | 1 | 1 | 16.0 |  | 50-250 mg/day |
| Geary | 1 | 1 |  | 30.0 | 6 mcg/min |
| Thacker | 2 | 2 |  | 27.5 | 6-7 mcg/min |
| Trilli | 1 | 1 | 24.0 |  | 8.5-9 mcg/min |
| Tovar | 1 | 1 | 50.0 |  | 5-15 mcg/min |
| Thomas | 1 | 0 |  | *^g^* | 5-20 mcg/min |
| Jackson | 1 | 0 | *^h^* |  | 3-18 mcg/min |
| Ryding | 1 | 1 |  | 18.0 | 3.5-4.2 mcg/min |
| Newby | 1 | 1 | 30.0 |  | 0.77-2.2 mcg/min |
| Wray | 1 | 0 |  | *^i^* | 8-22 mcg/min |
| **Total** | **57** | **28** | **86.6***^a^* | **25.8***^a^* |  |
|  |  |  |  |  |  |
| *^a^* Weighted Average  *^b^* Data unavailable | |  |  |  |  |
| *^c^*  From < 90 mmHg to > 90 mmHg | | | |  |  |
| *^d^*  From BP of 76/48 to Diastolic of > 68 mmHg | | | |  |  |
| *^e^*  20 mmHg increase from average SBP of 81.7 mmHg | | | | | |
| *^f^* From 52.8 mmHg to > 100 mmHg in 13 of 18 patients. | | | | | |
| *^g^*  From 52 mmHg to > 100 mmHg | | | |  |  |
| *^h^*  From 50 mmHg to > 100 mmHg | | | |  |  |
| *^I^*  From < 80 mmHg to > 80mmHg | | | |  |  |
