# Supplementary material for: The effect of angiotensin II on blood pressure in patients with circulatory shock: a structured review of the literature
Source: Crit Care. 2017 Dec 28;21:324. doi: 10.1186/s13054-017-1896-6 (PMC5745607; doi:10.1186/s13054-017-1896-6)
Supplement: Supplementary file 2 — Cardiac arrest, describes results of the Ang II effect associated with all patients identified by the authors as being in a state of cardiac arrest (i.e., BP of 0/0). (DOCX 13 kb) [file 13054_2017_1896_MOESM2_ESM.docx]

| **Table S2. Cardiac Arrest** | | | | |
| --- | --- | --- | --- | --- |
| **Author (*year*)** | **Cases** | **Increase in SBP** | **Responders / Nonresponders** | **Dose Range** |
|  |  |  |  |  |
| Del Greco (*1961*) | 5 | 63.2 | 3 / 2 | 1.2-120.0 mcg/min |
| Nassif (*1963*) | 4 | 156.3 | 4 / 0 | 120-1,500 mcg bolus, 15.0-83.3 mcg/min |
| Wedeen (*1963*) | 4 | 113.5 | 4 / 0 | 15.0-83.3 mcg/min |
| Beenlands (*1964*) | 5 | *^a^* | 3 / 2 | 1.5-36.0 mcg/min |
| **Total** | **18** | **107.3***^b^* | **14 / 4** |  |
|  |  |  |  |  |
| *^a^* Responded to >100 mmHg | | |  |  |
| *^b^* Weighted average of all patients for whom data exists | | | | |
