# Supplementary material for: The effect of angiotensin II on blood pressure in patients with circulatory shock: a structured review of the literature
Source: Crit Care. 2017 Dec 28;21:324. doi: 10.1186/s13054-017-1896-6 (PMC5745607; doi:10.1186/s13054-017-1896-6)
Supplement: Supplementary file 3 — Results after removal of all cardiac arrest patients, describes primary analysis after removal of all patients identified by the authors as being in a state of cardiac arrest (i.e., BP of 0/0). (DOCX 15 kb) [file 13054_2017_1896_MOESM3_ESM.docx]

| **Table S3. Results after Removal of All Cardiac Arrest Patients** | | | | |
| --- | --- | --- | --- | --- |
| **Author** | ***n*** | **Number of Cases with Complete Data** | **Increase in SBP** | **Increase in MAP** |
|  |  |  |  |  |
| Del Greco | 15 | 15 | 42.1 |  |
| Nassif | 10 | 10 | 76.5 |  |
| Wedeen | 7 | 3 | 38.0 |  |
| Beenlands | 12 | 0 | *^a^* |  |
| Udhoji | 12 | 6 |  | 34.3 |
| Belle | 1 | 1 | 16.0 |  |
| Geary | 1 | 1 |  | 30.0 |
| Thacker | 2 | 2 |  | 27.5 |
| Trilli | 1 | 1 | 24.0 |  |
| Tovar | 1 | 1 | 50.0 |  |
| Cohn (AIM) | 6 | 6 |  | 29.7 |
| Singh | 25 | 0 | *^b^* |  |
| Wallace | 7 | 7 |  | 22.9 |
| Thomas | 1 | 0 |  | *^c^* |
| Jackson | 1 | 0 | *^d^* |  |
| Ryding | 1 | 1 |  | 18.0 |
| Newby | 1 | 1 | 30.0 |  |
| Wray | 1 | 0 |  | *^e^* |
| Eyraud | 14 | 14 | 74.0 |  |
| Chawla | 10 | 10 |  | 6.0 |
| Cohn (JCI) | 22 | 22 |  | 22.1 |
| Sorensen | 8 | 0 | *^f^* |  |
| Moore | 9 | 0 | *^g^* |  |
| Khanna | 163 | 163 |  | 12.5 |
| **Total** | **331** | **264** | **58.0***^h^* | **14.8***^h^* |
|  |  |  |  |  |
| *^a^* From 73.1 mmHg to >100 mmHg in 10 of 13 patients. | | | | |
| *^b^* From < 90 mmHg to > 90 mmHg | | | |  |
| *^c^* From 52 mmHg to >100 mmHg | | | |  |
| *^d^* From 50 mmHg to >100 mmHg | | | |  |
| *^e^* From < 80mmHg to > 80mmHg | | | |  |
| *^f^*  From BP of 76/48 to Diastolic of > 68 mmHg | | | |  |
| *^g^*  20 mmHg increase from average SBP of 81.7 mmHg | | | | |
| *^h^* Weighted averages | |  |  |  |
