# Supplementary material for: The effect of angiotensin II on blood pressure in patients with circulatory shock: a structured review of the literature
Source: Crit Care. 2017 Dec 28;21:324. doi: 10.1186/s13054-017-1896-6 (PMC5745607; doi:10.1186/s13054-017-1896-6)
Supplement: Supplementary file 4 — Results with patients from Khanna et al. Rrmoved, describes primary analysis after exclusion of the patients from Khanna et al., which represent a proportionally large amount of the total patients included in the analysis. (DOCX 17 kb) [file 13054_2017_1896_MOESM4_ESM.docx]

| **Table S4: Results with Patients from Khanna et al. Removed** | | | | | | | | | | |
| --- | --- | --- | --- | --- | --- | --- | --- | --- | --- | --- |
| **Author** | ***n*** | **Cases with Complete Data** | **Type of Shock** | | | **Increase in SBP** | | **Increase in MAP** | | **Dose Range** |
|  |  |  | **Cardiogenic** | **Septic** | **Other** | |  |  |  | |
| Del Greco | 20 | 20 | 2 | 8 | 11 | | 47.4 |  | 0.23-100 mcg/min | |
| Nassif | 14 | 13 | 2 | 6 | 6 | | 106.9 |  | 7-1,500 mcg bolus, 5.3-68 mcg/min | |
| Wedeen | 15 | 7 | 11 | 1 | 3 | | 81.1 |  | 1.5-36 mcg/min | |
| Beenlands | 17 | 0 | 18 | 0 | 0 | | *^a^* |  | 1-36 mcg/min | |
| Udhoji | 12 | 6 | 3 | 4 | 5 | |  | 34.3 | *^b^* | |
| Belle | 1 | 1 | 1 | 0 | 0 | | 16.0 |  | 50-250 mg/day | |
| Geary | 1 | 1 | 1 | 0 | 0 | |  | 30.0 | 6 mcg/min | |
| Thacker | 2 | 2 | 0 | 0 | 2 | |  | 27.5 | 6-7 mcg/min | |
| Trilli | 1 | 1 | 1 | 0 | 0 | | 24.0 |  | 8.5-9 mcg/min | |
| Tovar | 1 | 1 | 0 | 0 | 1 | | 50.0 |  | 5-15 mcg/min | |
| Cohn (AIM) | 6 | 6 | 0 | 6 | 0 | |  | 29.7 | *^b^* | |
| Singh | 25 | 0 | 0 | 25 | 0 | | *^c^* |  | 4-12 mcg/min | |
| Wallace | 7 | 7 | 0 | 0 | 7 | |  | 22.9 | 0.75=3 mcg/min | |
| Thomas | 1 | 0 | 0 | 1 | 0 | |  | *^d^* | 5-20 mcg/min | |
| Jackson | 1 | 0 | 0 | 0 | 1 | | *^e^* |  | 3-18 mcg/min | |
| Ryding | 1 | 1 | 0 | 1 | 0 | |  | 18.0 | 3.5-4.2 mcg/min | |
| Newby | 1 | 1 | 0 | 0 | 1 | | 30.0 |  | 0.77-2.2 mcg/min | |
| Wray | 1 | 0 | 0 | 1 | 0 | |  | *^f^* | 8-22 mcg/min | |
| Eyraud | 14 | 14 | 0 | 0 | 14 | | 74.0 |  | 2.5 mcg bolus | |
| Chawla | 10 | 10 | 0 | 10 | 0 | |  | 6.0 | 15-20 ng/kg/min | |
| Cohn (JCI) | 22 | 22 | 0 | 0 | 22 | |  | 22.1 | 0.3-60 mcg/min | |
| Sorensen | 8 | 0 | 0 | 0 | 8 | | *^g^* |  | *^b^* | |
| Moore | 9 | 0 | 0 | 0 | 9 | | *^h^* |  | 30ng/kg/min | |
| **Total** | **190** | **113** | **38** | **62** | **90** | | **70.0***^i^* | **21.7***^i^* |  | |
|  |  |  |  |  |  | |  |  |  | |
| *^a^* From 52.8 mmHg to >100 mmHg in 13 of 18 patients. | | | | | | |  |  |  | |
| *^b^* Data unavailable | |  |  |  |  | |  |  |  | |
| *^c^* From < 90 mmHg to > 90 mmHg | | | |  |  | |  |  |  | |
| *^d^* From 52 mmHg to >100 mmHg | | | |  |  | |  |  |  | |
| *^e^* From 50 mmHg to >100 mmHg | | | |  |  | |  |  |  | |
| *^f^* From < 80 mmHg to > 80 mmHg | | | |  |  | |  |  |  | |
| *^g^* From BP of 76/48 to Diastolic of > 68mmHg | | | |  |  | |  |  |  | |
| *^h^* 20 mmHg increase from average SBP of 81.67 mmHg | | | | | | |  |  |  | |
| *^I^* Weighted averages | |  |  |  |  | |  |  |  | |
