# Supplementary material for: The effect of angiotensin II on blood pressure in patients with circulatory shock: a structured review of the literature
Source: Crit Care. 2017 Dec 28;21:324. doi: 10.1186/s13054-017-1896-6 (PMC5745607; doi:10.1186/s13054-017-1896-6)
Supplement: Supplementary file 5 — Septic shock results removing patients from Khanna et al., describes the analysis of Ang II n the septic shock sub-group after exclusion of the patients from Khanna et al., which represent a proportionally large amount of the total patients included in the analysis. (DOCX 13 kb) [file 13054_2017_1896_MOESM5_ESM.docx]

| **Table S5: Septic Shock Results Removing Patients from Khanna et al.** | | | | |
| --- | --- | --- | --- | --- |
| **Author** | **Number of Cases** | **Increase in SBP** | **Increase in MAP** | **Dose Range** |
| Del Greco | 7 | 29.3 |  | 0.23-50 mcg/min |
| Nassif | 6 | 73.3 |  | 10-120 mcg bolus, 2-15 mcg/min |
| Wedeen | 1 | 110.0 |  | 11-21 mcg/min |
| Udhoji | 4 |  | 40.5 | *^a^* |
| Cohn (AIM) | 6 |  | 29.7 | *^a^* |
| Singh | 25 | *^b^* |  | 4-12 mcg/min |
| Thomas | 1 |  | *^c^* | 5-20 mcg/min |
| Ryding | 1 |  | 18.0 | 3.5-4.2 mcg/min |
| Wray | 1 |  | *^d^* | 8-22 mcg/min |
| Chawla | 10 |  | 6.0 | 15-20 ng/kg/min |
| **Total** | **62** | **53.9*^e^*** | **19.9***^e^* |  |
|  |  |  |  |  |
| *^a^* Data unavailable | |  |  |  |
| *^b^* From < 90 mmHg to > 90 mmHg | | | |  |
| *^c^* From 52 mmHg to >100 mmHg | | | |  |
| *^d^* From < 80 mmHg to > 80 mmHg | | | |  |
| *^e^* Weighted averages | |  |  |  |
